# Exploring the diagnostic landscape of *Mannheimia haemolytica*: technologies, applications, and perspectives

**DOI:** 10.3389/fmicb.2025.1680478

**Published:** 2025-10-31

**Authors:** Chenxiao Wang, Xindong Bai, Juan Wang, Dongyang Ye, Leina Dou, Zengqi Yang

**Affiliations:** ^1^Department of Preventive Veterinary Medicine, College of Veterinary Medicine, Northwest A&F University, Yangling, China; ^2^Key Laboratory for Prevention and Control of Major Ruminant Diseases, Ministry of Agriculture and Rural Affairs, Yangling, China

**Keywords:** *Mannheimia haemolytica*, bovine respiratory disease, diagnostic methods, serological diagnosis, molecular diagnostics, new diagnostic technologies

## Abstract

*Mannheimia haemolytica* (*M. haemolytica*) is recognized as a primary etiological agent of bovine respiratory disease (BRD) and ovine contagious pleuropneumonia. The clinical burden associated with these infections highlights the importance of early diagnosis to enable timely therapeutic interventions and prevent large-scale outbreaks. Conventional diagnostic approaches, including culture-based isolation and biochemical identification, remain standard practices for *M. haemolytica* detection, which enable recovery of complete bacterial isolates for downstream analyses. Recent advances in molecular diagnostics technology have dramatically improved the sensitivity, specificity, and turnaround time of *M. haemolytica* detection. Immunological assays, including enzyme-linked immunosorbent assays and agglutination tests, are important for high-throughput screening in epidemiological investigations. Additionally, matrix-assisted laser desorption/ionization time-of-flight mass spectrometry has emerged as a valuable adjunct for the rapid, automated identification of *M. haemolytica*, further streamlining clinical workflows. While considerable progress has been made in diagnostic technologies for *M. haemolytica*, a comprehensive review of these methods remains lacking. Existing reviews largely focus on the broader BRD complex or pathogenesis, rather than systematically evaluating diagnostic strategies tailored to *M. haemolytica*. Therefore, we first to critically appraise and summarize recent developments in traditional, molecular, immunological, and mass spectrometric diagnostic techniques, providing a consolidated reference for early, accurate, and field-deployable detection of *M. haemolytica* infections.

## 1 Introduction

*Mannheimia haemolytica* (formerly *Pasteurella haemolytica*) is a Gram-negative, facultatively anaerobic bacterium, belong to the Pasteurellaceae family (Griffin et al., 2010). *M. haemolytica* is currently classified into 12 distinct serotypes (A1, A2, A5–A9, A12–A14, A16 and A17), as defined by antigenic variation in surface polysaccharides and outer membrane proteins (Mason et al., 2022). These serotypes persistently colonize the ruminant upper respiratory tract, particularly the nasopharynx and tonsillar crypts, as commensal microbiota (Highlander, 2001). Among them, serotypes A1 and A2 can colonize the upper respiratory tracts of both cattle and sheep. In clinically healthy cattle, serotype A2 predominates, while serotypes A2 and A6 are primarily associated with infections and disease manifestation in sheep. In contrast, serotype A1 is detected at relatively low frequencies (Gharib Mombeni et al., 2021). In the absence of external stressors, *M. haemolytica* persists as a commensal organism within the upper respiratory tract of ruminants. Perturbations like host stress particularly under long-distance transport or abrupt climatic shifts or co-infections may disturb this balance, facilitating the overgrowth of serotype A1 (Hodgins and Shewen, 2004). *M. haemolytica* is a well-known opportunistic and primary respiratory pathogen in sheep and lambs, where its overgrowth can lead to severe bronchopneumonia and high mortality, especially following stress or primary infection by agents such as parainfluenza virus or *Mycoplasma* spp. In sheep, *M. haemolytica* has also been implicated in acute mastitis and mammary gland necrosis; several studies report it as one of the most frequently isolated pathogens in clinical mastitis cases in lactating ewes, even surpassing *Staphylococcus aureus* (Arsenault et al., 2008; Omaleki et al., 2011). More importantly, *M. haemolytica* is a leading cause of severe Fibrinous pleuropneumonia and systemic infection, which is a globally prevalent and economically devastating disease in ruminant livestock, particularly cattle and sheep, with severe agricultural impact (Biesheuvel et al., 2021). Considering that the current vaccines only show limited serotype coverage and variable protective efficacy, the differential diagnosis from other respiratory pathogens causing nonspecific clinical presentation of infection is essential.

Despite widespread application, conventional biochemical identification methods exhibit critical limitations, including prolonged processing times and suboptimal sensitivity and specificity, making them insufficient for modern rapid diagnostic requirements (Confer and Ayalew, 2018). Recent advances in molecular diagnostics have revolutionized *M. haemolytica* detection. Fluorescence-based quantitative PCR (qPCR) assays targeting conserved genes (*ompA*, *16S rRNA*) achieve species-specific identification with high sensitivity in under 2 h—dramatically surpassing conventional culture-based methods in both speed and accuracy (Tabatabaei and Abdollahi, 2018). While qPCR offers superior diagnostic capabilities, its adoption is hindered by prohibitive costs and reliance on specialized laboratory equipment, particularly compromising its accessibility in low-resource settings (Dao et al., 2022). To overcome these challenges, loop-mediated isothermal amplification (LAMP) has emerged as a promising field-deployable solution, enabling equipment-independent detection with high analytical sensitivity (98.7%) and specificity (99.2%) within 60 min (Notomi, 2000). Complementarily, ELISA-based immunological assays provide valuable serological data through antibody detection, though their diagnostic window is constrained by the delayed humoral immune response, limiting sensitivity during early infection stages (Mohammad et al., 2023). Immunochromatographic techniques (ICTs) provide simple point-of-care testing tools, though further optimization is needed to enhance their diagnostic accuracy. Matrix-assisted laser desorption/ionization time-of-flight mass spectrometry (MALDI-TOF MS) has become a powerful tool for rapid bacterial identification, including potential applications for *Pasteurellaceae* characterization (Alispahic et al., 2011; Clark et al., 2013).

As demonstrated, researchers have made numerous effort for *M. haemolytica* diagnosis even if they carries various advantages and limitations in terms of accuracy, speed, cost, and practicality. The expanding diagnostic toolkit for *M. haemolytica* necessitates critical evaluation to guide optimal implementation and technological advancement. While previous reviews have focused on general respiratory pathogens or the broader bovine respiratory disease complex (BRDC), pathogen-specific diagnostic approaches for *M. haemolytica* remain underexplored (Kamel et al., 2024). This review provides a systematic summary and evaluation of current detection methods, detailing their working principles, specific applicability, and diagnosis performance for *M. haemolytica* identification (Table 1). In addition, we have systematically summarized the sensitivity, specificity, advantages, and limitations of the various methods (Table 2). Our analysis aims to inform clinical practice and drive innovation in diagnostic development. To our knowledge, this represents the first comprehensive review dedicated to *M. haemolytica* diagnostics, offering a timely resource to advance this important field of veterinary microbiology. This review systematically evaluates current diagnostic methods for *M. haemolytica*, providing insights to guide the development of improved detection technologies.

**Table 1:** Current detection methods of *M. haemolytica.*

Detection formats	Target	Sample source	Detection ranges	Assay time	References
Serological diagnostic technology					
Rapid plate agglutination	Antiserum	Bovine and Ovine	1:160	<10 s	Frank and Wessman (1978)
ELISA	Serum antibodies	Calves	1/1024	–	Opuda-Asibo et al. (1986)
Immunoperoxidase technique	*M. haemolytica*	lambs	–	–	Haziroglu et al. (1996)
Coagglutination test	*M. haemolytica*	Lung	–	–	Fodor et al. (1996)
Immunohistochemical	*M. haemolytica*	Lungs of cattle	–	<2 h	Yaman et al. (2018)
ELISA	IgG; IgM; IgA	Bovine	–	<3 h	Poonsuk et al. (2023)
Molecular diagnosis technology					
tDNA-PCR	tRNA	Sheep and Calf	–^a^	<2 h	Catry et al. (2004)
Real-time PCR	sodA	Field isolates	10^3^ bacterial cells per gram lung tissue sample	<5 h	Guenther et al. (2008)
Multiplex PCR	lkt	Bovine	>10 ng/reaction	<1.5 h	Alexander et al. (2008)
PCR	leukotoxin gene (lktA)	Ovine (Mastitis)	–	3 h	Omaleki et al. (2010)
PCR	gcp lktA	Bighorns (pneumonic lungs)	–	>1 h	Shanthalingam et al. (2014)
Multiplex PCR	Rpt2, PHSSA, 12S rRNA	Sheep lung	–	1.5 h	Kumar et al. (2015)
Multiplex PCR	ssa	poultry	–	1.5 h	Ammar et al. (2016)
One-run real-time PCR	sodA	Bovine nasal swab	<1 CFU	<1 h	Kishimoto et al. (2017)
RespoCheck PCR.	16 s rDNA V3	Calve lungs	>0.4 ~ 2 CFU/assay	>0.5 h	Wisselink et al. (2017)
Multiplex PCR	artJ lktC	Bovine	>40 pg./μL	1.5 h	Zhang et al. (2017)
Multiplex PCR-electronic microarray	lktA nmaA tbpB	Bovine respiratory	Most 1–10 copies Lowest 1,000 copies	2 h	Thanthrige-Don et al. (2018)
Multiplexed real-time PCR	lktCABD	Bovine lung tissue	>1.2 ~ 12 CFU/reaction	<1 h	Loy et al. (2018)
RPA	nmaA(A1 A6)	Bovine	>40 genome copies	30 min	Conrad et al., 2020
LAMP	rsmL	Bovine	>312 copies	<60 min	Pascual-Garrigos et al. (2021)
LAMP	rsmL/rsmC/lktA	Bovine nasal samples	>1,000 copies	45 min	Mohan et al. (2021)
Insulated isothermal PCR	sodA	Bovine and ovine respiratory	>21 copies for gDNA and 17.2 cfu/mL	<1 h	Dao et al. (2022)
LAMP	genotype 1 and 2 (Lkt D)	Bovine	>1–100 copies of targets	1 h	Dassanayake et al. (2023)
Multiplex PCR	Lkt	Bighorn sheep	>25–500 ng	>3 h	Fox et al. (2023)
Instrument diagnostic technology					
MALDI-TOF MS	ribosomal proteins	Bovine	–	–	Puchalski et al. (2016)
MALDI-TOF MS	genotypes 1 and 2	Bovine	–	–	Loy and Clawson (2017)
MALDI-TOF MS		Bovine	>1 × 10^7^–1 × 10^8^ CFU/mL	–	Van Driessche et al. (2019)

^a^Not mentioned in this work.

**Table 2:** Summary of sensitivity, specificity, advantages and limitations of major detection methods for *M. haemolytica.*

Method	Reported sensitivity (Se)	Reported specificity (Sp)	advantages	limitations	Representative references
Serology (ELISA, LFIA, LAT)	Often variable; examples: IgG ELISA ~ ~ 91% (study-specific). Sensitivity depends on isotype (IgG/IgM) and timing post-infection	Variable; example IgG Sp ≈ 87% (study-specific)	Inexpensive, high throughput, useful for herd-level surveillance and retrospective exposure assessment	Cannot reliably distinguish active vs. past infection; window period after infection; possible cross-reactivity depending on antigen design	Poonsuk et al. (2023)
Conventional PCR / multiplex PCR	Typically high for specific assays (commonly ~80–100% depending on primer/assay validation). Multiplex formats may show slightly reduced Se for some targets	Generally high (often ~98–100% for validated assays against panels of isolates)	High analytical sensitivity and specificity; can detect specific species/genes; multiplexing permits multi-pathogen screening	Multiplex efficiency may be limited for certain targets (some species require single-plex to reach same performance); labor and contamination risk; requires lab infrastructure	Loy et al. (2018) and Wisselink et al. (2017)
Real-time quantitative PCR (qPCR)	Very high; detection limits down to few copies; reported diagnostic Se frequently >90% in well-validated assays	Very high when assays are well designed and validated (often ~98–100% in reports)	Quantitative (Ct values), rapid, highly sensitive (low LOD), amenable to high throughput and automation	Requires specialized instrumentation, extraction steps; inhibitors in clinical samples can reduce performance	Kumar et al. (2019)
LAMP	Reported ranges in reviewed methods: ~66.7–100% (study dependent). Analytical LOD can approach ~1 copy/reaction in optimized assays	Reported ~95–100% for many assays in the literature (depends on primer design and validation)	Rapid, isothermal (no thermocycler), tolerant of crude samples, suitable for field/POC use, fast time-to-result	Primer design critical (risk of non-specific amplification); variable quantitative capacity; some assays show lower Se in complex matrices if primers/targets are suboptimal	Pascual-Garrigos et al. (2021)
Integrated / novel PCR	Comparable analytical Se to qPCR in some validations; LODs reported in low copy numbers (platform and assay dependent). Field validations report high Se/Sp for certain targets	High in validation studies (assay dependent)	Portable/field-deployable, automated readouts, faster workflow suitable for on-site testing	Still dependent on assay design; validation across varied clinical matrices is limited; cost of devices and consumables	
MALDI-TOF MS	For direct use on complex samples (e.g., BALf) reported Se is moderate (e.g., ~57% overall in mixed samples in one study); for cultured isolates Se is typically very high for species ID	For direct-from-sample applications Sp reported very high (100% in some studies); for isolates, excellent specificity when databases are adequate	Extremely rapid and high-throughput identification from pure isolates; low per-sample cost after instrument investment; reduced time vs. culture/phenotyping	Requires pure culture for routine reliable ID; direct application to complex clinical material is experimentally promising but limited by low microbial load and interfering substances; depends on quality/coverage of spectral databases	Van Driessche et al. (2019)

## 2 Serological diagnostic techniques

Traditional immunological diagnostic approaches have long been utilized in the diagnosis of BRD, primarily focusing on the detection of pathogen-specific antibodies or antigens. These tests rely on the principle of antigen–antibody interactions, enabling the identification of infections based on the host’s immune response. These methods are valued for their simplicity and high specificity, making them well-suited for large-scale screenings and on-site diagnostics.

**Figure 1:**
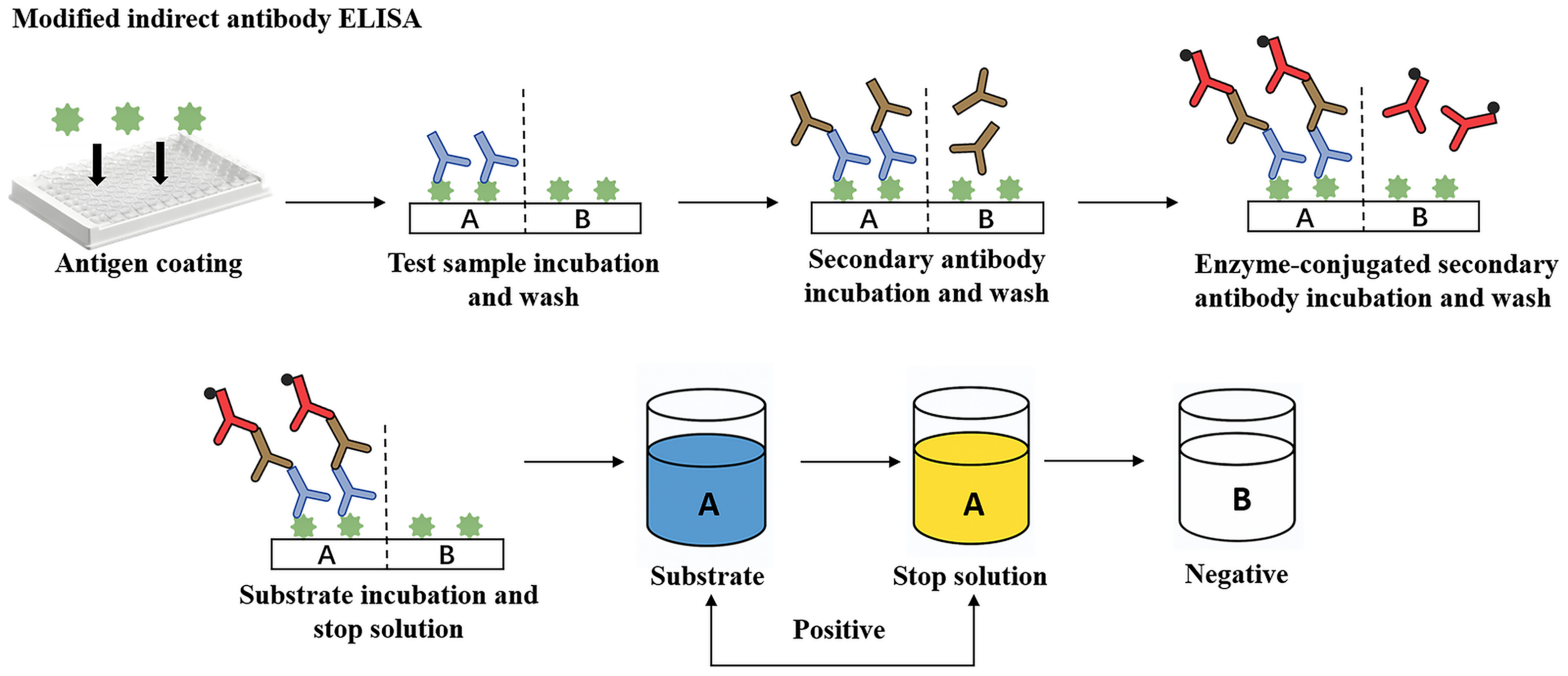
A modified indirect antibody ELISA for quantitation of class-specific antibodies (IgA, IgG, IgG2, IgM) against *M. haemolytica*. Green antigen = formalinized *M. haemolytica*; Blue antibody = test sera/BAW; Brown antibody = unlabelled rabbit anti-bovine globulins (anti-IgA, -IgGL, -IgG2, IgM); Red antibody = goat anti-rabbit HRPO-conjugates.

Frank and Wessman (1978) introduced a plate agglutination test for serotyping *M. haemolytica* strains. This method allows detection of bovine and ovine strains within 10 s, even when antigens are diluted up to 160 times, though it requires pre-prepared specific sera and has lower sensitivity compared to modern techniques. Subsequently, Opuda-Asibo et al. (1986) developed an improved ELISA method to detect *M. haemolytica*-specific antibodies in bronchoalveolar lavage fluids and sera from vaccinated cattle and sheep (Figure 1). This method achieves a minimum detection dilution of 1/1024, facilitates rapid screening of numerous samples, and reveals higher levels of IgG1, IgG2, and IgGM in sera, with IgG1, IgG2, and IgGA predominant in bronchoalveolar washings samples. Fodor et al. (1996) prepared agglutinin reagents using hyperimmune sera, enabling rapid detection of viable or dead pathogens in lung tissues. This approach identifies 94% of wild-type strain serotypes, maintains high specificity despite minor cross-reactions and auto-agglutination, and detects 36% of specific antigens in healthy sheep lung specimens. Additionally, Haziroglu et al. (1996) employed an extravidin-biotin-peroxidase complex method for immunoperoxidase staining of sheep lung tissue sections, successfully localizing *M. haemolytica* antigens primarily within neutrophils. Although low bacterial loads might lead to false negatives, this method excels in pinpointing lesions across different lung regions.

In recent years, immunohistochemical staining has emerged as a valuable tool for elucidating the spatial distribution and tissue tropism of *M. haemolytica* in the context of disease pathogenesis. Yaman et al. (2018) observed widespread distribution of *M. haemolytica* on alveolar epithelium, bronchi, and bronchioles in pneumonia cases naturally infected with Bovine Respiratory Syncytial Virus (BRSV), suggesting that BRSV infection might facilitate concurrent *M. haemolytica* infection. More recently, Poonsuk et al. (2023) developed an indirect ELISA method using whole-cell antigens to detect IgG, IgM, and IgA antibodies against *M. haemolytica* in BRD, completing tests within 3 h with 90% sensitivity and over 80% specificity. They noted that high levels of specific antibodies might reflect a protective state rather than active disease, emphasizing the need for additional diagnostic methods for definitive diagnosis.

In summary, traditional immunological methods are essential for large-scale screenings and preliminary on-site diagnostics. However, their sensitivity may be affected by factors such as sample quality and testing conditions, often necessitating their integration with molecular techniques to mitigate these limitations. When integrated with molecular biology techniques, these methods significantly enhance detection sensitivity and accuracy, thereby providing a robust technical foundation for comprehensive BRD diagnosis and effective disease management.

## 3 Molecular diagnostic techniques

**Figure 2:**
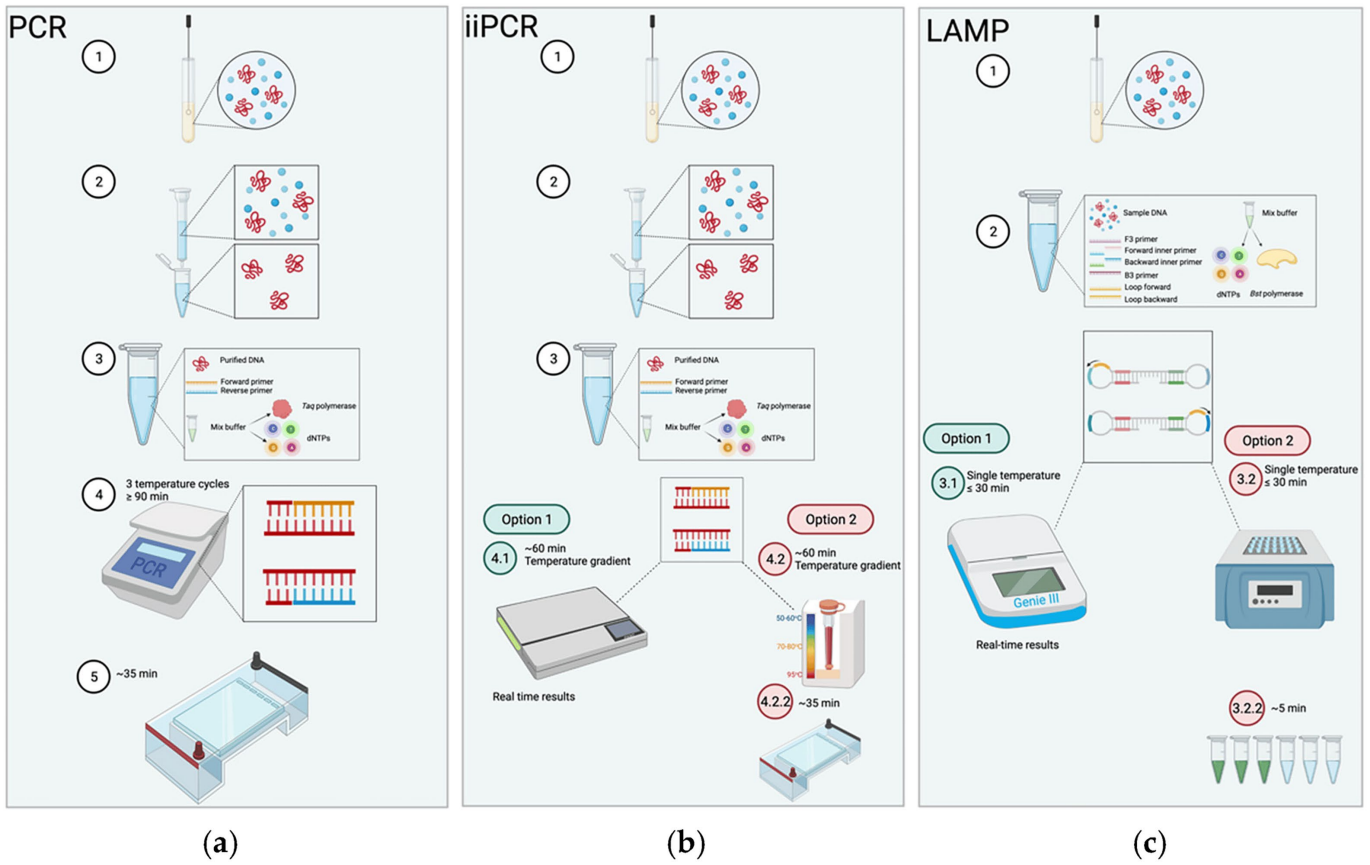
The procedures of PCR, insulated isothermal PCR (iiPCR), and LAMP. **(a)** Conventional PCR: After collected and purified, the sample is mixed with primers and master mix. The reaction undergoes thermal cycling (~90 min) and is followed by gel electrophoresis to visualize products. **(b)** iiPCR: After collected and purified, the sample is reacted under a thermal gradient (convection-driven isothermal amplification) for ~60 min, with results obtained either in real time or via gel electrophoresis. **(c)** LAMP: The collected sample is mixed with 4–6 primers directly (no purification needed); the mixture is incubated at a constant temperature (≤30 min) with detection achieved through real-time monitoring or simple visual signal (e.g., fluorescence/color change). Adapted from Knox and Beddoe (2021) *Animals*, licensed under CC BY 4.0. https://creativecommons.org/licenses/by/4.0/.

Since introduction in the late 1980s, polymerase chain reaction (PCR) technology has revolutionized molecular diagnostics, establishing itself as a foundational tool in pathogen detection and disease management (Saiki et al., 1985; Schmitz et al., 2022). Over the following decades, the emergence of advanced molecular techniques—such as multiplex PCR, real-time quantative PCR (RT-qPCR), loop-mediated isothermal amplification(LAMP), recombinase polymerase amplification (RPA) and insulated isothermal PCR (iiPCR)—has significantly enhanced diagnostic sensitivity, specificity, and speed (Figure 2; Subsoontorn et al., 2020; Liu et al., 2023). These innovations have substantially improved the detection performance of *M. haemolytica* and other respiratory pathogens, enabling faster and more accurate diagnosis (Bell et al., 2014). The integration of diverse molecular platforms has expanded diagnostic capabilities beyond conventional laboratory settings, supporting the development of portable, point-of-care systems, and offering vital support for timely intervention and effective disease control (Krishna and Cunnion, 2012; Qian et al., 2020).

*Mannheimia haemolytica* deploys a repertoire of virulence factors that orchestrate host colonization, immune evasion, and tissue damage. Central to its pathogenic potential is the leukotoxin operon (lktCABD), which encodes a potent member of the Repeats-in-Toxin (RTX) family that selectively targets ruminant leukocytes, playing a pivotal role in disease pathogenesis (Davies et al., 2002; Aulik et al., 2010). In addition, genes such as *P. haemolytica* serotype-1 specific antigen (PHSSA) and *Rpt2* have been identified as serotype-specific markers, facilitating precise strain differentiation. The superoxide dismutase A (sodA) gene, encoding superoxide dismutase A, is highly conserved across *M. haemolytica* isolates and serves as a robust species-specific molecular signature (Davies et al., 1997). Moreover, adhesin-related genes—including the adhesin pseudogene B1 and adhesin G—have been linked to genotype-level variability, shedding light on the species’ genetic diversity. Collectively, these genetic determinants not only illuminate the molecular underpinnings of *M. haemolytica* pathogenesis but also represent crucial targets for the development of sensitive and specific molecular diagnostics, thereby advancing the precision of clinical pathogen detection.

### 3.1 PCR and multiplex PCR

Early implementations of singleplex PCR offered rapid and precise detection of *M. haemolytica*. Catry et al. (2004) pioneered a PCR assay targeting the tRNA intergenic spacer, enabling differentiation between Pasteurella and Mannheimia genera within 2 h, foundational for ensuing molecular advancements. Shanthalingam et al. (2014) developed a PCR assay targeting the *gcp* gene, enabling direct detection of the pathogen from lung tissues of deceased sheep. Even in culture-negative cases, this method yielded a PCR-positive rate of 77%, further confirming the decisive role of target gene selection in detection sensitivity and accuracy. In ovine mastitis diagnostics, Omaleki et al. (2010) combined *LktA*, *rpoB*, and *rrnA* gene amplification with phylogenetic analysis to confirm isolates in approximately 3 h.

To meet heightened demands for throughput and efficiency, multiplex PCR techniques were adopted. Alexander et al. (2008) introduced assays targeting HP, Lkt, Lkt2, and 16S rRNA for differentiating *M. haemolytica*, *M. glucosida*, and *M. ruminalis* using as little as 10 ng DNA in about 1.5 h. Kumar et al. (2015) extended the strategy by targeting *PHSSA* and *Rpt2* genes and incorporating sheep mitochondrial 12S rRNA as an internal control, enhancing both accuracy and reliability. Within the context of BRDC, Zhang et al. (2017) developed a multiplex PCR targeting *KMT1* (*P. multocida*), *artJ-lktC* (*M. haemolytica*), and *plo* (*T. pyogenes*), achieving a low detection limit of 40 pg./μL within 1.5 h. In parallel, Ammar et al. (2016) developed a multiplex PCR assay capable of detecting six avian respiratory pathogens simultaneously. While four targets were reliably detected in multiplex format, *M. haemolytica* and *P. multocida* still required single-plex PCR. Furthermore, they observed that targeting *Lkt* confers greater specificity than *ssa*, emphasizing the critical role of target gene selection in diagnostic assay development. For addressing the diagnostic complexity in wild ruminants, Fox et al. (2023) developed an integrated culture-independent platform combining multiplex PCR, amplicon sequencing, and bioinformatics. Simultaneously detecting *M. haemolytica*, *B. trehalosi*, *P. multocida*, and *M. ovipneumoniae* via lktA and 16S rRNA analysis, this approach enables multilocus sequence typing and high-resolution data interpretation even a complex workflow employing 38 primer pairs was entailed.

Together, advances in both singleplex and multiplex PCR methodologies have dramatically reduced diagnostic turnaround—from roughly 2 h to 1.5–3 h—while enhancing sensitivity (detection limit down to 40 pg./μL) and specificity. The integration of sequencing and multilocus analysis further extends diagnostic precision, forming a seamless continuum from rapid screening to definitive identification that overcomes limitations inherent to traditional culture-based diagnostics and bolsters both clinical and epidemiologic utility.

### 3.2 Real-time quantitative PCR

Real-time quantitative PCR (RT-qPCR) has emerged as a cornerstone in pathogen detection and identification due to its high sensitivity, specificity, and rapid turnaround time. Unlike conventional PCR, RT-qPCR not only provides real-time quantitative results that help in accurately assessing pathogen load, but also delivers quicker feedback for clinical treatment support. For *M. haemolytica*, RT-qPCR methods have been continually refined and are now applied in monitoring diseases in poultry, cattle, and other animals.

Guenther et al. (2008) developed a RT-qPCR assay targeting a conserved region of the *sodA* gene, enabling the rapid and species-specific identification of five Mannheimia species—*M. haemolytica*, *M.* var*igena*, *M. ruminalis*, *M. granulomatis*, and *M. glucosida*—within 5 h, with a detection sensitivity of approximately 10^3^ bacterial cells per gram of lung tissue. Their findings highlighted the superior discriminatory power of the *sodA* gene over the highly conserved *16S rRNA* gene, underscoring its utility as a robust molecular marker for interspecies differentiation within the Mannheimia genus. Building on prior advancements, Wisselink et al. (2017) developed a multiplex RT-qPCR platform—RespoCheck PCR—specifically optimized for the simultaneous detection of four major BRD pathogens: *P. multocida*, *M. haemolytica*, *Histophilus somni*, and *Trueperella pyogenes*, using bronchoalveolar lavage fluid as the diagnostic sample. The assay demonstrated exceptional analytical sensitivity, detecting as little as 1–10 fg of purified DNA per reaction, and achieved a diagnostic specificity of 98.3%. Notably, the detection of the *M. haemolytica* V3 region within the 16S rDNA was completed in approximately 30 min, underscoring the assay’s potential as a rapid and reliable tool for early-stage BRD diagnosis. Furthermore, Loy et al. (2018) established a multiplex RT-qPCR assay employing hydrolysis probes and validated its application on both Peltier and rotary thermal cycler platforms for the detection of key pathogens implicated in the BRDC. The assay specifically targets four contiguous genes within the leukotoxin operon (lktCABD), enabling robust pathogen identification. Comparable diagnostic performance was observed across platforms, with a sensitivity of 80.5% and specificity of 88.8% on the Peltier system, and 80.1% sensitivity and 88.3% specificity on the rotary system. The assay achieved detection limits ranging from 1.2 to 12 CFU per reaction, representing a significant improvement in analytical sensitivity and diagnostic efficiency over other comparable approaches. In addition, Kishimoto et al. (2017) introduced an integrated, single-run RT-qPCR system for the comprehensive detection of 16 pathogens associated with BRDC, encompassing 10 viral and 6 bacterial agents. By specifically targeting the highly conserved *sodA* gene of *M. haemolytica*, the assay enabled highly specific identification of this pathogen within a diagnostic timeframe of less than 1 h, achieving a detection limit as low as 1 CFU per reaction. By targeting a conserved genetic region and minimizing assay duration, the risk of sample degradation was substantially reduced, leading to marked improvements in diagnostic sensitivity and specificity in this work.

Overall, the evolution of RT-qPCR techniques—from single-target assays to multi-platform integrated systems—has led to significant improvements in detection times (low as 0.5 h) and lower detection limits (from 10^3^ bacterial cells per gram to as low as 1 CFU per reaction). Although each method has its specific focus, collectively they form a highly efficient, precise, and sensitive diagnostic system that not only compensates for the limitations of singleplex PCR and Multiplex PCR method but also provides robust technical support for the early diagnosis and clinical treatment of respiratory diseases.

### 3.3 Loop-mediated isothermal amplification

Loop-mediated isothermal amplification (LAMP) offers a highly promising molecular diagnostic method for point-of-care detection of *M. haemolytica*. It’s detection limit can reach approximately one copy per reaction. The described LAMP assays achieved sensitivities between 66.7 and 100% and specificities between 95 and 100%, while retaining exceptional simplicity of operation under isothermal conditions. Unlike conventional PCR, LAMP does not rely on complex thermal cycling equipment, making it particularly well suited for rapid, on-site diagnostics in resource-limited settings. In recent years, LAMP assays have garnered increasing attention for the detection of pathogens implicated in BRDC, offering a robust, inhibition-resistant alternative for rapid and reliable clinical diagnostics.

**Figure 3:**
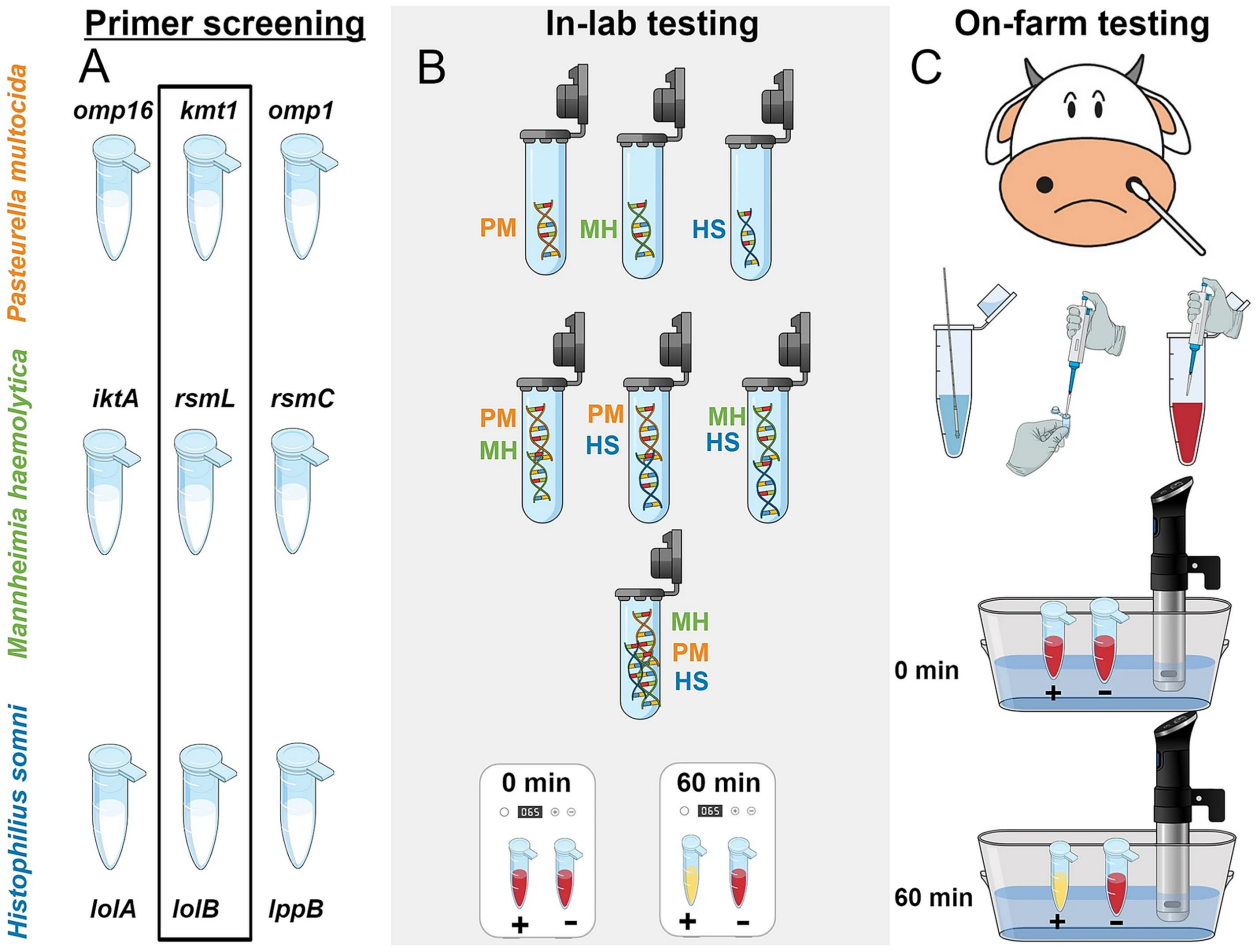
Overall schematic of the workflow of LAMP assay constructed by Pascual-Garrigos et al. (2021). **(A)** Three different primers were screened through the limit of detection (LOD) study. The best-selected primers in each species were highlighted inside the black rectangle. **(B)** Several combinations of DNA were diluted in water and tested in the lab environment to study off-target behavior in pH-sensitive colorimetric reactions. **(C)** LAMP was conducted on-farm with a prepared colorimetric master-mix and later repeated in-lab. A precision cooker was used as a heating device to confirm the ability of the test in a resource-limited setting. PM, *Pasteurella multocida*; MH, *Mannheimia haemolytica*; HS, *Histophilus somni* (Adapted from Pascual-Garrigos et al. (2021) Veterinary Research, licensed under CC BY 4.0. https://creativecommons.org/licenses/by/4.0/).

Mohan et al. (2021) developed a LAMP assay targeting three major BRD pathogens—*M. haemolytica*, *P. multocida*, and *H. somni*. They designed three sets of primers targeting the *rsmL*, *rsmC*, and *lktA* genes of *M. haemolytica*, and after a series of experimental optimizations, identified the optimal primer combination that enabled detection to be completed within 45 min. The assay achieved detection limits of 10^3^ copies/reaction in water samples and 10^4^ copies/reaction in liquid Amies samples, demonstrating simplicity of operation and straightforward result interpretation, which makes it highly suitable for rapid field diagnosis. Building on this, Pascual-Garrigos et al. (2021) further developed a LAMP method for detecting *P. multocida*, *M. haemolytica*, and *H. somni* in BRD (Figure 3). By employing a pre-formulated colorimetric master mix combined with instrument-based detection, this method achieved high analytical sensitivity and specificity, allowing for clear visual detection within 60 min and reaching a detection limit of 312 copies per reaction. However, the assay performed poorly in detecting *M. haemolytica*, as false negatives were observed due to cross-reactivity with other off-target DNA. This outcome underscores that the selection of target genes and the screening of primers remain critical challenges that must be addressed in future research. Furthermore, Dassanayake et al. (2023) further refined LAMP technology by establishing a genotypically discriminative assay capable of differentiating between genotype 1 and genotype 2 strains of *M. haemolytica* associated with BRDC. They selected the adhesin pseudogene B1 for genotype 1 and adhesin G for genotype 2 as targets, and designed highly specific LAMP primers that allowed for detection within 1 h. The assay demonstrated detection limits ranging from 1 to 100 copies per reaction and effectively avoided cross-reactivity with other members of the Pasteurellaceae family. Moreover, their study noted that the LAMP primers targeting the *rsmL* gene used by Mohan et al. exhibited insufficient specificity, further emphasizing the importance of precise target selection and primer design in clinical applications.

Collectively, these studies underscore the substantial potential of LAMP technology for the detection of BRD pathogens. With detection times ranging from 45 to 60 min and sensitivity extending from 10^3^ copies per reaction to as low as 1 copy per reaction, these methods are undergoing continuous optimization and serve as complementary diagnostic tools. Beyond field-applicable precision diagnostics, these methods provide a roadmap for improving clinical sensitivity, minimizing diagnostic errors, and resolving strain-specific pathogenicity.

### 3.4 Integrated/novel PCR methods

Continuous breakthroughs in molecular biology technology have led to the quick emergence of innovative PCR methods, greatly expanding the approaches and procedures for detecting BRD infections. Conventional PCR techniques, recognized for their exceptional sensitivity and specificity, are highly effective in identifying individual infections. Nonetheless, when addressing intricate samples that harbor many pathogens concurrently, numerous tests are frequently necessary, rendering the process both time-consuming and labor-intensive. In order to resolve this matter, researchers have been investigating novel approaches that integrate multiplex PCR with other molecular diagnostic technologies (Zhang et al., 2015). These approaches facilitate the concurrent identification of several pathogens in a single reaction, while also markedly decreasing detection time, improving sensitivity, and minimizing reliance on intricate instrumentation, thereby demonstrating potential uses in field diagnostics.

**Figure 4:**
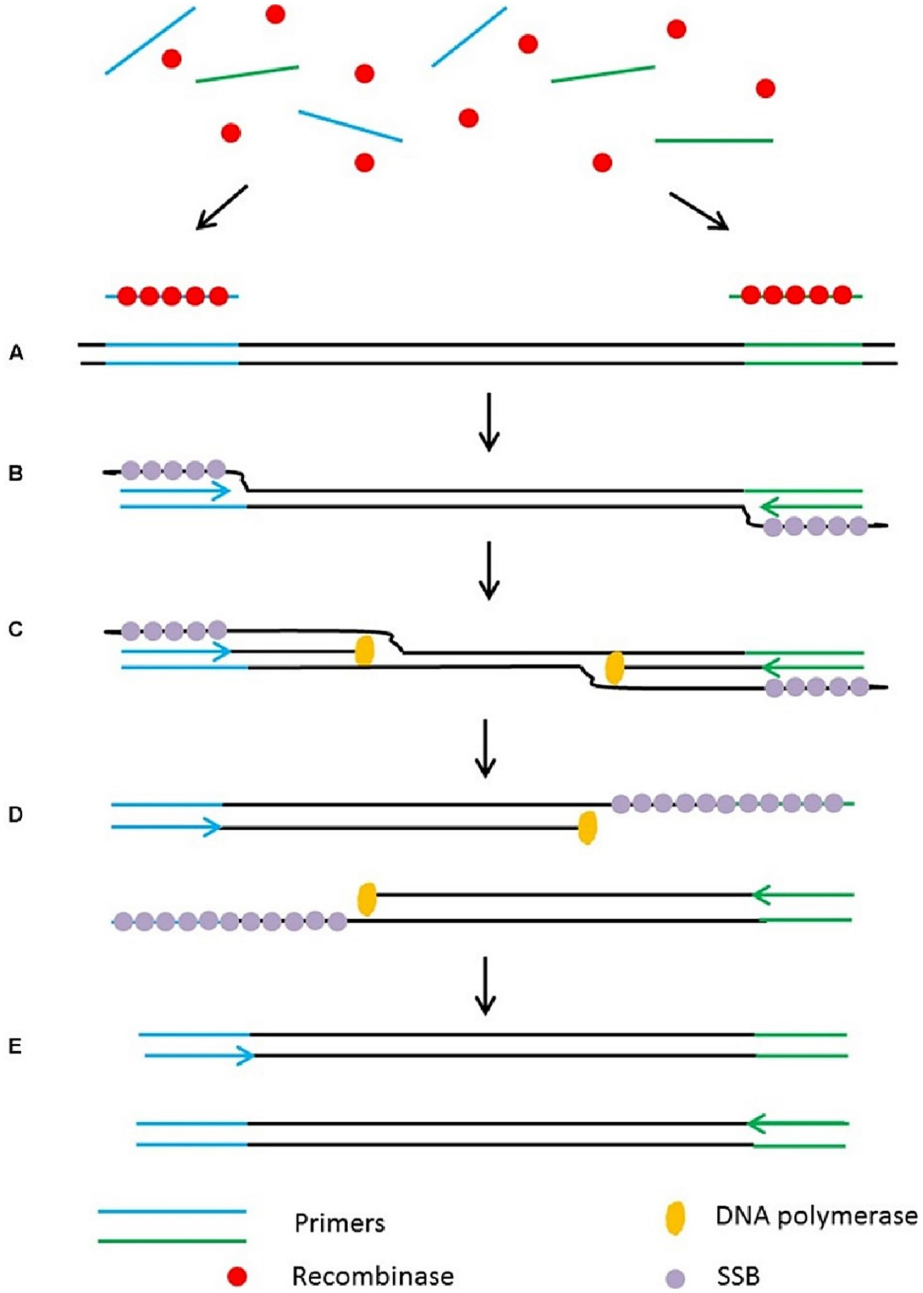
Schematic outline of the recombinase polymerase amplification (RPA). **(A)** Recombinase integrates with primers to form recombinase-primer complexes and target specific DNA sequences. **(B)** Strand exchange occurs and single stranded binding proteins (SSB) bind to the DNA to form a D-loop. **(C)** DNA polymerase initiates DNA amplification. **(D)** Displaced D-loop stabilized by SSB as amplification continues. **(E)** Two dsDNA molecules form and the entire cycle start again. Adapted from Lau and Botella (2017), *Frontiers in plant science*, licensed under CC BY 4.0. https://creativecommons.org/licenses/by/4.0/.

Thanthrige-Don et al. (2018) integrated multiplex PCR technologies with DNA microarray to simultaneously detect four bacterial pathogens (*M. haemolytica*, *H. somni*, *P. multocida*, *M. bovis*) and five viral pathogens (such as Bovine Parainfluenza Virus Type 3, Bovine Respiratory Syncytial Virus, etc.) of BRD. The detection can be completed within 2 h, with a detection limit as low as 1–10 copies, demonstrating high sensitivity and specificity. Recombinase polymerase amplification (RPA) offers rapid, ultra-sensitive detection at constant low temperatures (37–42 °C), enabling amplification of as few as 1–10 target DNA copies within 10–20 min using minimal equipment, making it exceptionally suited to field-deployable or point-of-care diagnostics (Figure 4). Conrad et al. (2020) designed and validated 11 RPA detection methods, ultimately developing a scheme suitable for detecting four BRD pathogens (*M. haemolytica*, *P. multocida*, *H. somni*, *M. bovis*), antimicrobial resistance genes (AMR), and integrative conjugative elements (ICE) simultaneously. Targeting the *nmnA* gene of *M. haemolytica* serotypes A1 and A6, the method achieved a detection limit of 40 genomic copies, with sensitivity comparable to RT-qPCR. Since it does not rely on complex instrumentation, results can be obtained within 20–30 min, making it highly suitable for rapid field diagnostics. Additionally, Dao et al. (2022) developed a insulated isothermal PCR (iiPCR) method, specifically designed for the *sodA* gene of *M. haemolytica*. This method achieved a detection limit of 21 genomic copies or 17.2 cfu/mL of bacterial culture, offering a 10 to 100-fold increase in sensitivity over traditional PCR methods, and can complete detection within 1 h. This method combines rapid turnaround (≤ 1 h), a simplified portable platform based on thermal convection, and sensitivity comparable to nested PCR—enabling robust, field-compatible pathogen diagnostics.

These novel PCR methods ingeniously combine techniques such as multiplex amplification, microarray detection, RPA, and isothermal PCR, which achieved decreased detection times (low as 20–30 min) and LOD from (low as 1–10 copies), demonstrating significant improvements in efficiency and sensitivity. These methods complement each other, collectively forming an efficient, flexible, and field-adaptable pathogen detection system. This system provides a solid technological foundation for the early diagnosis, precise treatment, and prevention of BRD, while also pointing the way forward for future optimization and standardization of detection processes.

To date, diagnostic efforts for *M. haemolytica* have largely centered on the molecular techniques outlined in this chapter, such as PCR and its variants. Although next-generation sequencing (NGS) is becoming commonplace in veterinary microbiology, its application for the direct and specific detection of *M. haemolytica* remains notably underrepresented. One notable exception is the study published by Anis et al. (2018) deployed a panel of 198 primers targeting 43 bovine pathogens, including *M. haemolytica*, and successfully detected these pathogens in samples with qPCR Ct values in the 30s. However, beyond this proof-of-concept implementation for syndromic NGS panels, the use of NGS for *M. haemolytica* has primarily been limited to comparative genomics, serotype/genotype classification, and broader phylogenetic analyses, rather than being applied as a routine diagnostic method (Sahay et al., 2019).

## 4 Matrix-assisted laser desorption/ionization time-of-flight mass spectrometry

In recent years, MALDI-TOF MS has demonstrated exceptional potential as a high-throughput and accurate platform for microbial identification, offering significant advancements in clinical microbiology and diagnostic precision. By analyzing characteristic protein fingerprints of bacteria, MALDI-TOF MS can accurately identify pathogens at the species level. Studies have shown that this method has up to 100% consistency with traditional microbial identification techniques (Klima et al., 2014). More importantly, MALDI-TOF MS can detect differences between various genotypes of *M. haemolytica*, helping to rapidly distinguish strains in cattle affected by BRD (Puchalski et al., 2016; Loy and Clawson, 2017). Notably, Van Driessche et al. (2019) first applied MALDI-TOF MS to rapidly detect bovine respiratory pathogens directly from bronchoalveolar lavage fluid samples. The detection limit for *M. haemolytica* reached 1 × 10^7 to 1 × 10^8 CFU/mL. Compared to traditional culture methods, this technique showed 73% detection consistency, sensitivity greater than 75, and 100% specificity with no false positives.

Beyond species identification, MALDI-TOF MS can differentiate between *M. haemolytica* genotypes—namely, the virulent genotype 2 and commensal genotype 1—enabling precise differentiation relevant to BRD pathogenesis and facilitating targeted downstream diagnostics (Loy, 2020; Loy et al., 2023). The process include obtained a protein mass fingerprint from a cultured colony, compared with a reference database, and delivered results in minutes (Calderaro and Chezzi, 2024). However, effective implementation of MALDI-TOF MS still faces several challenges. First, MALDI-TOF MS requires a pure culture isolate — although its direct application to complex clinical materials such as bronchoalveolar lavage (BAL) fluid has shown experimental promise, this approach is not yet routine in diagnostic settings, largely because low microbial burdens and interfering host or environmental substances often impair reliable identification. Second, database coverage poses limitations: reference libraries heavily skew toward human pathogens and may lack adequate entries for veterinary or rare BRD-associated strains, which undermines accuracy (Rychert, 2019; Han et al., 2021). Furthermore, although methods such as MBT-ASTRA aim to extend MALDI-TOF into antimicrobial resistance detection, these remain largely experimental and are not yet standard practice (Oviaño and Bou, 2019).

Overall, MALDI-TOF MS, based on ribosomal protein identification, provides an economical and rapid method for identifying *M. haemolytica*, which is crucial for confirming infections. Additionally, mass spectrometry holds promise for large-scale epidemiological testing, helping to understand the role of *M. haemolytica* in BRD and laying the foundation for effective prevention strategies.

## 5 Practical considerations and method selection

### 5.1 Sample collection and quality control

Accurate detection of *M. haemolytica* across diagnostic modalities depends critically on appropriate sample collection, careful preservation, and rigorous quality control. Nasal swabs are minimally invasive and easily obtained in the field, but they frequently contain mixed microbial flora that make results interpretation complicated. In contrast, bronchoalveolar lavage fluid and lung tissue samples—though more invasive and requiring stricter biosafety measures—typically yield higher pathogen loads and more clinically relevant information, thereby enhancing diagnostic sensitivity. Maintaining sample integrity is essential to ensure reliable results across various diagnostic modalities. Specimens should be stored at 4 °C for short-term use and frozen at −80 °C for long-term preservation to prevent degradation of nucleic acids, proteins, and antigens. Inadequate temperature control or processing delays will significantly diminish assay performance (Puchalski et al., 2016). In the detection process, laboratories must implement comprehensive internal and external quality control systems. The routine use of internal controls is indispensable: positive controls (e.g., well-characterized *M. haemolytica* strains) verify assay performance, while negative controls (e.g., blank matrices) help detect contamination or procedural errors across various workflows. For molecular diagnostics, spatial separation of pre- and post-amplification areas, along with the use of dedicated equipment, and protective apparel, is critical to prevent amplicon contamination (Toohey-Kurth et al., 2020).

Furthermore, adherence to CLSI M58 guideline (2017) can ensure that tests are fit for purpose and perform reliably across contexts. This includes daily instrument calibration, routine verification using standardized reference materials, and validation within laboratory-specific contexts to ensure accurate pathogen identification and classification (Rau et al., 2022). For MALDI-TOF MS, compliance with standards such as ISO 16140-3 and CLSI M58 is vital.

Finally, systematic documentation is essential for supporting traceability and reproducibility. Recording detailed metadata—such as animal species, patient ID, sample type, collection time, storage conditions, tests process, and operator information—are beneficial for analyzing correlation across assays, supporting audit processes, and ensuring accurate interpretation (Flatland et al., 2010).

### 5.2 Context-driven diagnostic pathways

Selecting the optimal diagnostic strategy for *M. haemolytica* hinges on balancing clinical urgency, resource availability, and the depth of information required. In field or low-resource environments, such as farms or remote veterinary stations, isothermal amplification methods like RPA and LAMP offer clear advantages. RPA can operate at a constant temperature (37–42 °C) even using rudimentary heat sources such as body warmth and yield results within 20 min with minimal equipment, making it a prime candidate for rapid, point-of-care applications (Tan et al., 2022; Munguti et al., 2024). LAMP excels advantages of rapid (<60 min), robust amplification, and minimal instrumentation needs, achieving high sensitivity and specificity even in the presence of sample inhibitors (Das et al., 2022). While in well-equipped laboratories, PCR-based modalities, particularly real-time quantitative PCR (qPCR) and insulated isothermal PCR (iiPCR) are remain a gold standard. These methods deliver high specificity and sensitivity, allow quantification of pathogen load, and enable simultaneous detection of multiple targets, making them indispensable when diagnostic accuracy and throughput are paramount. For advanced strain-level distinctions, such as differentiating virulent genotype 2 from commensal genotype 1 of *M. haemolytica*, a culture followed by MALDI-TOF MS offers rapid and informative results. MALDI-TOF MS can distinguish these genotypes through distinct protein mass signatures, providing critical epidemiological and pathogenicity insights. However, MALDI-TOF MS are not yet ready for routine clinical deployment even if demonstrated potential, faster turnaround and streamlined spectrum analysis. For therapeutic decisions require antibiotic stewardship, antimicrobial susceptibility testing (AST) of traditional culture-based MIC testing is remain the definitive method (Wilhelm et al., 2023).

In summary, a pragmatic decision-making pathway may be articulated as follows: for on-site, rapid screening, RPA or LAMP is ideal; for accurate laboratory-based confirmation, PCR or iiPCR is preferred; for genotypic and epidemiological insights, culture plus MALDI-TOF MS is invaluable; and for guiding antimicrobial therapy, culture-based AST remains the benchmark. This tiered framework equips practitioners to match diagnostic approaches to specific clinical contexts and resource constraints, enhancing both effectiveness and efficiency.

## 6 Outlook

With the advancement of molecular biology techniques, traditional culture and biochemical identification methods are gradually being replaced by PCR, RT-qPCR, and multiplex PCR. While these technologies have significantly improved in sensitivity and specificity, false negatives or false positives are still occured in low-load or complex samples. And traditional PCR has longer reaction times and higher sample quality requirements. At the same time, although high-throughput sequencing can provide comprehensive and precise genomic information, its daily application remains limited due to high costs and complex operations. More importantly, existing research remains predominantly centered on *M. haemolytica* isolates from cattle, with substantial gaps in target selection, detection limit optimization, and clinical validation for ovine-derived strains. These limitations present critical challenges to advancing diagnostic precision and enhancing disease management strategies in sheep populations. Efforts should focus on improving the sensitivity, specificity, and clinical feasibility of detection methods, particularly for the establishment and optimization of detection methods for sheep-derived *M. haemolytica*.

First, the detection efficiency of PCR and qPCR can be further enhanced by optimizing primer design, improving reaction systems, and refining sample processing (e.g., using nanotechnology or magnetic particles). Optimizing qPCR detection targets and developing multiplex PCR for simultaneous detection of *M. haemolytica* and its associated pathogens (e.g., *Pasteurella multocida* and *Bibersteinia trehalosi*) will improve diagnostic accuracy. Additionally, integrating emerging molecular diagnostic techniques like nanotechnology, microfluidic chips, and CRISPR-Cas could further shorten detection times, reduce costs, and enable rapid on-site detection.

Secondly, with the advancement of bioinformatics and artificial intelligence, MALDI-TOF MS technology is expected to improve the detection sensitivity for low-abundance samples through automation, and in combination with high-throughput sequencing, enable rapid analysis of large sample sets. Future research should focus on optimizing sensitivity (e.g., through immunocapture technology), expanding database coverage, and promoting the standardization of MS-AST, thereby enhancing its application in complex infection diagnostics and precision treatment.

Finally, system biology analysis based on whole-genome data will provide new insights into pathogens differentiate, offering new perspectives for accurate diagnosis. Furthermore, the integration of machine learning and artificial intelligence will significantly improve the analysis efficiency, optimizing automated identification processes, facilitating large-scale epidemiological investigations, and pathogen tracking. However, its clinical translation remains limited, primarily due to constraints related to instrumentation requirements, operational time, and associated costs.

Thus, when selecting detection methods, it is essential to consider pathogen load, clinical needs, laboratory equipment, time, and cost, as each technique has its advantages and disadvantages. On the other hand, we must recognize that while the rapid advancement of diagnostic technologies holds great promise for early detection, it concurrently gives rise to pressing societal concerns—including data privacy and disparities in access due to the digital divide. In addition to safeguarding the security of genomic and clinical data, measures must be taken to and address potential inequities in resource distribution during the dissemination of these technologies. How to rationally utilize these technologies while ensuring privacy security, avoid data misuse and over-interpretation, and balance economic feasibility and industrial promotion, making these detection methods affordable and usable by grassroots veterinarians and farmers, will be a significant challenge in the future of veterinary public health.

In summary, molecular detection enabling early and accurate *M. haemolytica* identification and advancing clinical diagnostics, epidemiological surveillance, and precision veterinary medicine, lay the foundation for more effective, targeted interventions. Future innovation should focus on translating these tools into routine practice, especially in complex and resource-limited settings, by convergencing of cutting-edge platforms, including nanotechnology, microfluidics, CRISPR-Cas systems, and artificial intelligence to overcome current limitations in sensitivity, scalability, and usability. The seamless integration of molecular diagnostics with bioinformatics and intelligent systems will be pivotal in realizing next-generation, high-throughput detection frameworks that can dynamically support veterinary decision-making and global health strategies.

## 7 Conclusion

Detection methods for *M. haemolytica* have evolved from traditional bacterial culturing and biochemical identification to molecular biological techniques, including PCR/RT-qPCR, LAMP, and MALDI-TOF MS, significantly enhancing detection sensitivity, specificity, and speed. Distinct advantages and limitations of each available diagnostic modality were summarized and analyzed in this review. As well as the future research of these diagnoise technologies also put forward. It is envisioned that this review will serve as a foundational reference to inform clinical practice, guide future research, and accelerate the development of next-generation, pathogen-specific diagnostics for *M. haemolytica*, thereby contributing to improved disease control in veterinary medicine and public health.
